# Do Dipolar Cosolvents Mitigate Microheterogeneity in Deep Eutectic Solvents? Solvation Dynamics and Solute Rotations in Glyceline/Methanol Solutions

**DOI:** 10.1021/acs.jpcb.5c06534

**Published:** 2025-12-28

**Authors:** Christian Green, Christopher A. Rumble, Mark P. Heitz

**Affiliations:** † Department of Chemistry and Biochemistry, 14788 SUNY Brockport, Brockport, New York 14420, United States; ‡ 43296 The Pennsylvania State University − Altoona College, 3000 Ivyside Park, Altoona, Pennsylvania 16601, United States

## Abstract

Steady-state and time-resolved fluorescence was used to investigate the solvation and rotational dynamics of coumarin 153 (C153) and coumarin 343 (C343) in binary solutions of methanol and glyceline, a deep eutectic solvent (DES) composed of a 1:2 molar ratio of choline chloride and glycerol. Time-resolved Stokes shifts were used to quantify the solvation dynamics and were found to be biexponential for both C153 and C343 with nearly identical integral solvation times. The solvation times were also found to be weakly dependent upon solution viscosity, and a power law exponent of *p* = 0.18 was found for mole fractions of glyceline greater than *x*_DES_ = 0.2. Solute rotational dynamics were explored using time-resolved fluorescence anisotropy. The reorientation times of C153 and C343 were found to be single exponential at all mixture compositions but did not follow a power-law dependence on solution viscosity. Instead, there was evidence for preferential solvation of the probes by components of glyceline.

## Graphical abstract

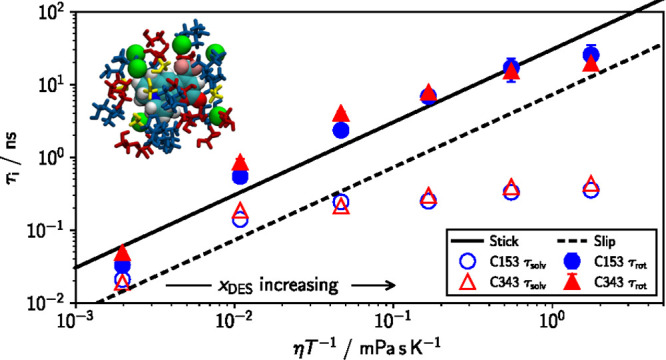

## 1 Introduction

Among the most extensively studied deep eutectic solvents (DESs) to date, type III DESs are formed by mixing a quaternary ammonium salt as a hydrogen bond acceptor (HBA) with a molecular hydrogen bond donor (HBD). Further description of all types I–V is available for the interested reader.^1,2^ The classic examples are choline chloride (ChCl, HBA) mixed with ethylene glycol, glycerol, or urea as the HBD, termed ethaline, glyceline, and reline, respectively. These ‘canonical’ DESs^3^ are composed of a 1:2 mol ratio of HBA: HBD. Another broad category of DESs is the natural deep eutectic solvent (NADES), first reported by Choi,^4^ are touted for their ‘greenness’ due to their biodegradability, biocompatibility, and sustainability.^5^ With respect to using the term ‘deep eutectic’ itself, there is an ongoing debate about what the proper, technical definition is and what exactly constitutes such a solvent. However, it is beyond our purpose here to delve into this debate. Although reports of DES usage have proliferated since the mid-2000s, applications have generally outpaced studies of their fundamental chemistry and physics. Continued interest in these solvents has spawned an increased number of systematic investigations into structure, interactions, dynamics, and solvation, which have led to a deeper understanding and more insightful and selective applications.

As DESs continue to gain prominence in literature reports, interactions between DES components have been more closely scrutinized through molecular dynamics simulations,^6–11^ neutron scattering,^12–16^ and NMR,^17–20^ unveiling the underlying complexity and heterogeneity. Other experimental studies have used time-resolved spectroscopy to measure the solvation and rotation dynamics of fluorescent dyes as a means of assessing DES spatial and temporal heterogeneity.^21–29^ For example, Samanta and co-workers observed spatial and dynamic heterogeneity in the solvation response of quaternary ammonium salt DESs such as choline chloride and tetraalkylammonium bromide.^24^ They observed two time regimes as represented by a subpicosecond time constant accompanied by a slower time constant on the order of several hundred picoseconds. The longer relaxation time was attributed to a heterogeneous diffusional motion, while the shorter time was from a faster librational type relaxation.^24,25^ Biswas and co-workers studied a biodegradable NADES comprised of glucose-urea using coumarin 153 (C153) dye to record rotation and solvation dynamics.^26^ In these experiments, it was shown that the NADESs also showed two time constants, but at ∼100 ps and a few ns. Here, the slower time was due to the high viscosity of the medium, whereas the ∼100 ps reflected the dynamics of the constituents in the nano- and microscopic domains with the NADES microenvironment. Investigations of the composition-dependent reorientation dynamics in several DESs have also been very recently reported by Blanchard and colleagues, who used time-resolved fluorescence anisotropy of several fluorophores to examine the rotational reorientation kinetics in type III DESs.^27–29^ In glyceline, they observed that cationic (oxazine 725) and neutral (perylene) dyes were more significantly decoupled from DES viscosity than was the anion dye (rose bengal), and all showed a significant increase in reorientation time at 15 mol % (∼1:5.7 ChCl:Gly component ratio). In addition, these dyes also displayed sub-slip hydrodynamic behavior, especially at the smaller composition ratios, and approached the slip limit as the ratio approached 33 mol % (1:2 ChCl:Gly component ratio). Dependence on composition ratio revealed a coupling length scale dependence that was greater than what the chromophore microenvironment would adequately probe. These few examples clearly show the variety of responses that can occur in DESs and therefore underscore the importance of a case-by-case evaluation of DES systems and interactions within.

The structure and dynamics of neat DESs have been the primary focus in much of the DES literature, with the inclusion of cosolvents in recent years. Like ILs, DES viscosity can be high^30,31^ such as with neat glyceline at ∼500 mPa s at 25 °C, and for applications it may be advantageous to modify the viscosity by adding a molecular cosolvent. Studies of cosolvents as DES diluents have focused more on water than on other molecular solvents, and relatively fewer studies have discussed effects from alcohols or other dipolar solvents.

Much of the cosolvent work has described the physicochemical properties and thermodynamics of the DES/cosolvent systems.^30,32–36^ Wang et al.^30^ reported the effects of mixing methanol and water with glyceline and ethaline by measuring density and viscosity. Excess molar volumes in glyceline and ethaline both showed negative deviations from ideal mixing, with excess molar volume minima at *x*_DES_ ≈ 0.35 for methanol and *x*_DES_ ≈ 0.40 for water that were explained as significantly attractive intermolecular interactions among the solution constituents. Viscosity deviation minima were observed at much larger DES mole fractions, *x*_DES_ = 0.70 for methanol and *x*_DES_ = 0.60 for water, and by all measures, glyceline exhibited a greater effect over ethaline because of the additional −OH. Further, methanol was observed to have a greater effect on solution properties than did water. Similar observations were reported by Agieienko and Buchner^31^ for glyceline using DMSO as the cosolvent, where the excess molar volume minimum occurred at a DES composition *x*_DES_ = 0.42. Below this value, they suggested that volume contraction was from the solvation of glyceline components in DMSO cavities and at higher DES mole fraction DMSO resided in DES voids. Viscosity deviations showed a maximum at *x*_DES_ ≈ 0.55, typical of strong hydrogen bond interactions, which is less than what is reported for methanol. Evidently, interactions between alcohol and glyceline mitigate viscosity changes until a larger DES mole fraction is reached.

In addition to experiments, computer simulations have been used to study structural and dynamical changes in DESs as a function of cosolvent addition. For example, molecular dynamics (MD) simulations of ChCl/lactic acid/water demonstrated that the DES could be considered a nonregular confinement medium wherein water could occupy solvent cavities or voids.^37^ There was a clear water concentration distinction near 2000 ppm water, where the structure of the DES changed. For water concentrations below 2000 ppm, water was confined to voids in the DES and only interacted by hydrogen bonding and not by disturbing the surrounding solvent. However, for water concentrations above 2000 ppm, aggregates formed with sizes larger than the voids in the DES, necessitating a change in the DES structure.^37^ MD simulations of glyceline/water reported that the excess molar volume minimum was observed at *x*_DES_ ≈ 0.50, but it was noted that the simulations overestimate the nonideality of mixing.^38^ One other point of note in that report was that glycerol hydrogen bonded with up to four water molecules at higher water concentrations. The overarching point from these representative computational and experimental studies is that as cosolvent is added, thereby diluting the DES there is a maximal effect at *x*_DES_ = 0.30–0.50 depending on the specific DES composition.

While there has been an increase in literature reports that have used extrinsic solutes to probe DES behavior as a function of HBA and HBD composition, temperature, etc., there are fewer reports that have focused on DES/cosolvent composition, and of these, most have targeted water as the cosolvent. Given that DESs find a myriad of applications in modern chemical processes, such work is important to gain a fuller understanding of how cosolvents modify the DES behavior. Thus, it is our interest here to examine the impact of DES solution behavior on two solute molecules, or, inversely, how solute molecules report on DES compositional variation. We use the fluorescence from two coumarin probes, C153 and C343, to capture the energetic and dynamic characteristics of solvation in glyceline/methanol mixtures that span the range of solution mole fraction. We chose methanol as our cosolvent for this study for a number of reasons. It is chemically similar to HBD glycerol and may be expected to decrease viscosity without changing the polarity of the mixture. Additionally, it can be removed more easily by vacuum methods. Steady-state absorption and fluorescence emission spectra from each probe were used to determine the spectral response to variations in DES solution composition. We then present time-resolved emission spectra (TRES) used to elucidate the solvation dynamics as the glyceline content is varied. Finally, from anisotropy measurements, we examine each solute’s reorientation dynamics in solution by considering Stokes–Einstein–Debye (SED) theory to evaluate the observed dynamics against simple hydrodynamics.

## 2 Methods

### 2.1 Materials and Sample Preparation

Coumarin 153 (C153) and coumarin 343 (C343) were purchased from Exciton, stored under desiccation, and used as received. Methanol (MeOH) was HPLC graded from Fisher Scientific (USA). Glyceline was prepared using a 2:1 mol ratio of glycerol (ThermoScientific, USA, ultrapure spectrophotometric grade 99.5+%) to choline chloride (ChCl, Acros Organics, USA, 99%). ChCl was dried under a vacuum for 48 h prior to mixing with glycerol. The mixture was stirred at 60 °C for 24 h and dried on an evacuated Schlenk line at 60 °C overnight to remove moisture. A Mettler Toledo (USA) C20 Karl Fischer autotitrator with a DM 143-SC double platinum pin electrode was used to determine the water content of the solvents prior to the spectroscopic experiments and was found to be 120 ± 2 ppm in MeOH and 500 ± 25 ppm in glyceline. Measurements were performed at 295 K in at least triplicate. Molecular structures for all compound used in this work are shown in Figure 1.

**Figure 1:**
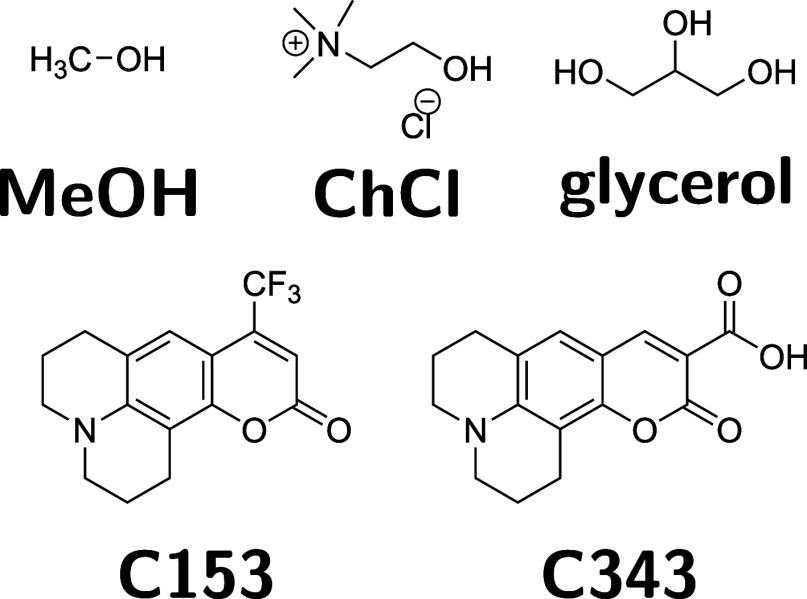
Molecular structures for all species used in this study.

### 2.2 Fluorescence Spectroscopy

Steady-state absorption was measured with a PerkinElmer Lambda 800 UV–vis spectrometer with a bandpass of 2 nm. Fluorescence excitation and emission spectra were acquired on a Horiba Scientific Fluorolog-3 spectrometer. Excitation was by a 450 W Xe arc lamp with wavelength selection through a single grating excitation monochromator and emission was acquired through a double subtractive grating emission monochromator with a spectral band-pass of 2 nm. Photons were detected by a TBX-850 photomultiplier tube. Calibration was performed daily by using the Raman signal from a sample of deionized water and the S/N ratio was >5000. All absorption and emission spectra were solvent subtracted and the emission spectra were corrected for wavelength-dependent instrument response using a NIST lamp generated correction file.

Broadband time-resolved fluorescence measurements were made using a femtosecond streak camera spectrometer coupled with a Ti:sapphire oscillator. Excitation was achieved using a Spectra-Physics Mai Tai femtosecond oscillator (Spectra-Physics, Milpitas, CA, USA) that produced a 2.93 W average power at 850 W at a repetition rate of 80 MHz and a fundamental pulse fwhm of ∼80 fs. The fundamental beam was fed into a GWU (Erftstadt, Germany) Ultrafast Harmonic Generator (UHG) unit that uses a Bragg cell pulse selector to reduce the 80 MHz pulse train to 80 kHz, and then frequency doubled using a BBO crystal to generate 425 nm photons for sample excitation. The average doubled output power was ∼200 mW or 2.5 nJ per pulse. Prior to entering the sample, the beam was passed through a half-wave plate followed by a Glan-Laser polarizer and focused onto the sample with a 100 mm focal length plano-convex lens. Emission from the sample was collected, collimated, and passed through a matching Glan-Laser polarizer set at a magic angle polarization (54.7°) with respect to excitation. A polarization scrambler was placed after the analyzing polarizer to eliminate any possible polarization-dependent sensitivity in the streak camera. A focusing lens then steered the emission into a SpectroPro 150 mm spectrograph (Teledyne Princeton Instruments, Trenton, New Jersey, USA). The output of the spectrograph was focused into a Hamamatsu universal streak camera Model C10910 outfitted with the M10912 fast plugin electronics (Hamamatsu, USA). Spectrograph and streak slits were set at 200 and 40 μM, respectively, to optimize both spectral intensity and time-resolution. Various other table optics were purchased from Newport Corporation (North Logan, UT, USA).

Polarization anisotropy measurements were performed with the emission polarizer rotated to 0° (parallel) and 90° (perpendicular) relative to the excitation beam. Optical polarization sensitivities of the spectrograph output were determined by measuring the instrument G factor. With the use of the polarization scrambler, replicate, independent measurements yielded a polarization sensitivity G factor between 0.97 and 1.03.

Time-resolved emission spectra (TRES) were collected over different time ranges within the streak camera’s electronics to optimize time-resolution while still measuring the complete solvation response or anisotropy decay. The instrument response function (IRF) for a particular measurement was determined by using the fwhm of the scattering of the excitation beam from a dilute glycogen solution. According to the manufacturer, the expected IRF is nominally 1% of the acquisition time range. In practice, however, electronic jitter becomes progressively more impactful as faster measurement time ranges are selected. For measurements reported in this work, data were acquired with time ranges between 500 ps and 20 ns, where the associated IRF widths found to be ∼10 and ∼200 ps, respectively. Electronic drift was also detected in the measurements, the effect of which was minimized using a sequence acquisition approach built into the camera software. After the acquisition of a complete data set, a built-in alignment algorithm adjusted each acquired sequence to compensate for the drift. The broadband spectra were corrected for wavelength-dependent detection sensitivities by reference to a set of secondary emission standards.^39^

## 3 Results

### 3.1 Steady-State Electronic Spectroscopy

We begin by examining the steady-state electronic absorption and emission spectra of C153 and C343 in the glyceline/MeOH mixtures displayed as a function of the absorption/emission wavelength in Figure 2. The C153 data exhibit broad, featureless, spectra expected for this dipolar probe in polar environments.^40^ A slight red-shift is observed in both sets of C153 spectra when transitioning from neat MeOH (*x*_DES_ = 0, blue lines) to neat glyceline (*x*_DES_ = 1, red lines), indicating that glyceline presents a slightly more polar environment than MeOH. There is little variation in the width of these spectra with changing solvent. This similarity is not unexpected, as both MeOH and the glyceline contain a high density of hydroxyl moieties which can participate in hydrogen bonding.

**Figure 2:**
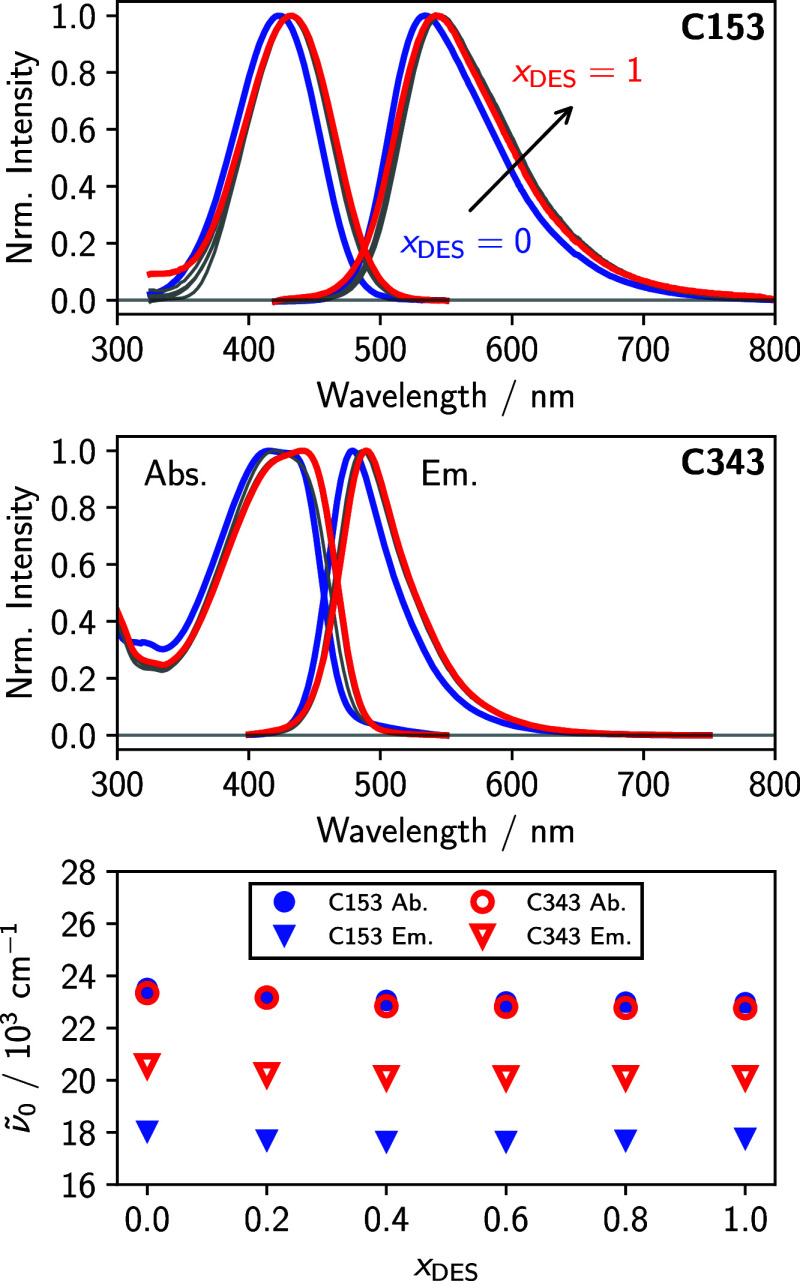
Normalized steady-state electronic absorption and emission spectra for C153 (top) and C343 (middle) in MeOH/glycleine mixtures. The bottom panel shows peak frequencies from fits to the absorption and emission lineshapes of C153 and C343 in the same mixtures.

The C343 spectra are also weakly dependent on the solvent. Emission spectra of C343 are significantly narrower than those for C153, but are still featureless. On the other hand, small changes in the shape of the absorption spectra are observed as the system increases in glyceline concentration. C343 possesses a smaller dipole moment change upon excitation than C153,^41^ therefore, its vibronic features are more prominent and their changes report on the changing solvent environment. Additionally, the carboxyl group on C343, which replaces the −CF_3_ on C153, can participate in hydrogen bonding, and this interaction can also alter the vibronic structure of its absorption spectrum.

To quantitatively describe the spectra, we first convert the absorption and emission spectra into the lineshape representation:

$$\begin{array}{c} \begin{array}{c} a(\overset{\sim }{\nu })\propto {\overset{\sim }{\nu }}^{-1}A(\overset{\sim }{\nu }) \end{array}\\ f(\overset{\sim }{\nu })\propto {\overset{\sim }{\nu }}^{-3}F(\overset{\sim }{\nu }) \end{array}$$ 1

where *A*(ν̃) and *F*(ν̃) are the absorption and emission spectra as a function of the wavenumber ν̃, and *a*(*ν̃*) and *f*(*ν̃*) are the respective absorption and emission lineshapes. This procedure removes the frequency dependence of the radiative rate from the spectra, making them directly proportional to the excited state population.^42^ The lineshape spectra for C153 and C343 are given in Figure S1 of the Supporting Information. We then fit the lineshapes to a log-normal function:

$$\begin{array}{c} l(\overset{\sim }{\nu })=h\{\begin{array}{ll} \exp[-\ln(2)\{(1+\alpha )\gamma {\}}^{2}], & \alpha (\overset{\sim }{\nu })>-1\\ 0, & \alpha (\overset{\sim }{\nu })\leq -1 \end{array}\\ \alpha (\overset{\sim }{\nu })=\frac{2\gamma (\overset{\sim }{\nu }-{\overset{\sim }{\nu }}_{0})}{\sigma } \end{array}$$ 2

where *l*(ν̃) is the absorption or emission lineshape, *h* is the height parameter, γ is the parameter related to the band asymmetry, ν̃_0_ is the peak frequency of the spectrum, and σ is a width parameter. We do not characterize the spectra with σ directly, but with Γ, the spectral full-width at half-maximum (fwhm):

$$\Gamma =\frac{\sigma \sinh(\gamma )}{\gamma }$$

The values of ν̃_0_ from the fits are shown in the bottom panel of Figure 2, and all fit parameters are given in Table 1. These data again demonstrate that both C153 and C343 experience similar solvation environments throughout the mixtures. The Stokes shifts, Δν̃ = ν̃^ab^ – ν̃^em^, for C153 are significantly larger than for C343 due to its larger excited state dipole moment, but the similar absorption ν̃_0_ for both probes indicates they have a similar ground state dipole moment. The time dependence of the Stokes shift will be our measure of the solvation dynamics in these systems and will be the focus of the next section of this work.

**Table 1:** Fitting Parameters of the Steady-State Lineshapes of C153 and C343 Fit to Eq 2 ^a^

	C153 absorption			C153 emission			
*x*_DES_	ν̃_0_	Γ	γ	ν̃_0_	Γ	γ	Δν̃
0.0	23.52	4.05	0.294	18.04	3.69	–0.308	5.48
0.2	23.12	4.01	0.279	17.70	3.67	–0.294	5.41
0.4	23.06	3.83	0.195	17.65	3.72	–0.307	5.41
0.6	23.00	3.94	0.254	17.65	3.65	–0.271	5.35
0.8	22.99	3.95	0.249	17.69	3.67	–0.263	5.30
1.0	22.97	4.24	0.341	17.77	3.87	–0.301	5.41

	C343 absorption			C343 emission			
*x*_DES_	ν̃_0_	Γ	γ	ν̃_0_	Γ	γ	Δν̃
0.0	23.34	5.04	0.911	20.56	3.00	–0.411	2.78
0.2	23.17	5.21	0.805	20.22	3.07	–0.398	2.94
0.4	22.84	5.24	0.907	20.12	3.06	–0.391	2.72
0.6	22.81	5.08	0.861	20.10	3.05	–0.386	2.71
0.8	22.77	5.13	0.889	20.11	3.07	–0.374	2.66
1.0	22.76	5.23	0.915	20.11	3.06	–0.375	2.64

a The quantity Δν̃ is the difference between the absorption and emission peak frequencies, or Stokes shift. Values of ν̃_0_, Γ, and Δν̃ are given in units of 10^3^ cm^–1^ and γ is a unitless quantity.

### 3.2 Solvation Dynamics

The solvation dynamics of the mixtures were measured using time-resolved emission (TRE) spectra collected with the aforementioned streak camera system. Traditionally,^40^ the goal of such experiments is to measure the solvation response, *S*(*t*), through measurement of the normalized time-dependent emission peak frequencies. One then calculates *S*(*t*) according to

$$S(t)=\frac{{\tilde{\nu }}_{0}(t)-{\tilde{\nu }}_{0}(\infty )}{{\tilde{\nu }}_{0}(0)-{\tilde{\nu }}_{0}(\infty )}$$ 3

where ν̃_0_(*t*) is the time-dependent peak emission frequency, and ν̃_0_(0) and ν̃_0_(*∞*) are the peak frequencies of before any solvent relaxation and at equilibrium, respectively. Their difference, the denominator of eq 3, then represents the total time-resolved Stokes shift of the probe. The value of ν̃_0_(*∞*) can be easily determined from the position of the TRE spectra after the shift has been completed. On the other hand, the time-zero position, ν̃_0_(0), must be calculated from steady-state absorption and emission spectra under the assumption that the absorption spectrum of the probe in a nonpolar reference solvent has the same vibronic character as in the target polar solvent.^43^ While this assumption holds for C153, it does not hold for C343 due to the changing vibronic character of the C343 spectra as a function of mixture composition (Figure 2). Therefore, for the sake of consistency, we will not determine *S*(*t*) in this work, but simply:

$$s(t)={\tilde{\nu }}_{0}(t)-{\tilde{\nu }}_{0}(\infty )$$ 4

This function will report on the same dynamics as *S*(*t*), but will not be normalized to the total shift. In effect, we ignore the portion of the shift that occurs before the resolution of our instrument. Therefore, the solvation times measured here represent the upper bound of their true values.

Before examining *s*(*t*), we pause to discuss how the TRE spectra from the streak camera are collected and processed. As previously discussed, the time resolution of the streak camera is dependent upon the time window used for collection. Because the solvation response for these solvents is significantly faster than the excited state lifetimes of the probes, we choose for each solvent the smallest time window that covers the entire solvation response. This maximizes the time resolution while still capturing all of the solvation dynamics. Sample TRE spectra and emission decays for C153 in neat MeOH with a 500 ps window are shown in the top panels of Figure 3, and for C153 in neat glyceline with a 20 ns window in the bottom panels of the same figure. Also included for reference are the IRFs for each measurement.

**Figure 3:**
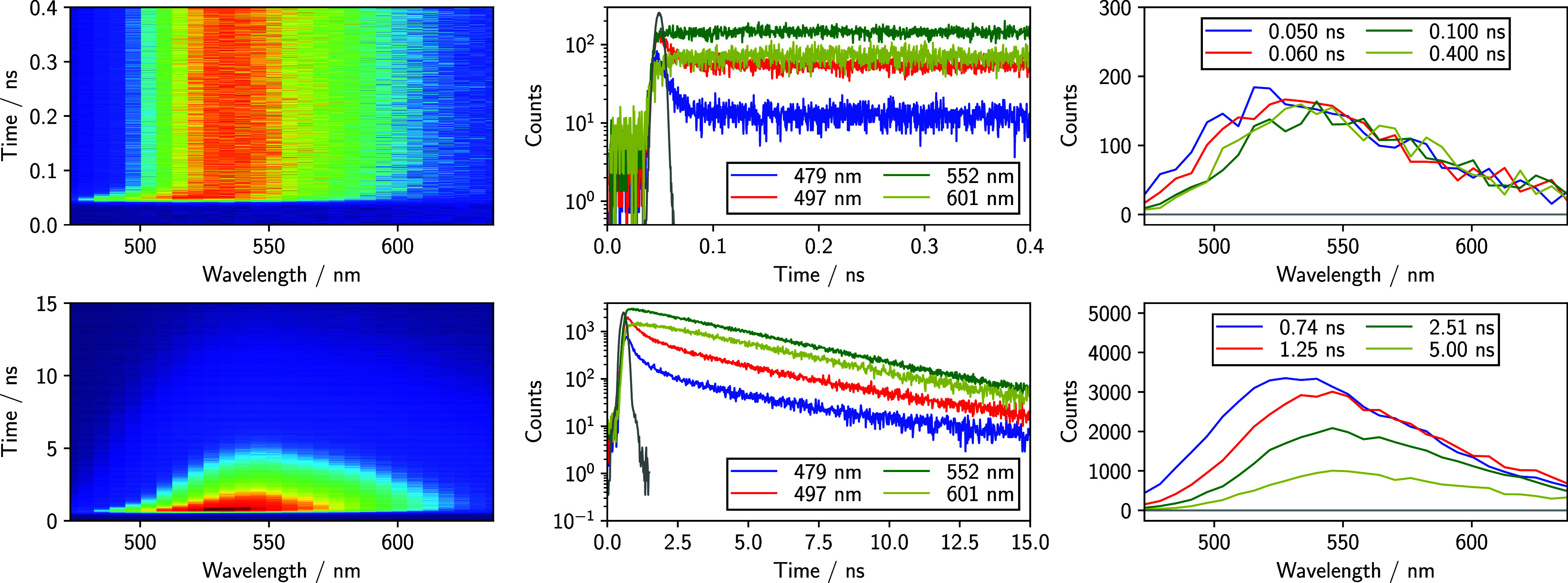
Upper set of three panels: C153 in neat MeOH using the 500 ps time range at 295 K. In the emission matrix (left panel), blue is lower intensity and red is higher intensity. Decays for several representative wavelengths (middle panel) were extracted from the 2-D matrix. The gray line is the instrument response function (IRF). Time-resolved emission spectra (right panel) are from representative time delays following excitation. Lower set of three panels: same descriptions as above, but for neat C153 in neat glyceline at 295 K using the 20 ns time range. Legends specify the wavelength and time details for each plot on the respective graphs. Note that the laser pulse time delay was ∼50 ps (gray line, middle panel), and the initial emission intensity is observed just after excitation. Thus, for the MeOH data, the 0.050 ns spectrum (blue line) approximates what would be the time-zero emission spectrum.

The next step in processing the TRE spectra is to deconvolute the effect of instrumental broadening, which is accomplished using a convolute-and-compare least-squares fitting routine. We model the TRE intensity at a particular wavelength, *I*(*t*, λ), by a sum of exponentials:

$$I(t,\lambda )=\sum_{i=1}^{n}a_{i}(\lambda )\exp(-t/\tau_{i})$$ 5

where *a*_*i*_(λ) is the amplitude of component *i* and τ_*i*_ the corresponding time-constant. Our fitting algorithm allows *a*_*i*_(λ) to vary with wavelength, but the time-constants are shared across all wavelengths. This method is effectively the same as global analysis^42,44^ but without the matrix division as the exponential function must be convoluted with the instrument function with a wavelength-dependent time-zero shift of the IRF. Example fit results are shwon in Figures S2 and S3 of the Supporting Information.

The resulting fitted exponential parameters are then used to construct what we term the ‘ideal’ deconvoluted probe spectrum. These data can then be used to extract *s*(*t*) by fitting each ideal spectrum to the log-normal function given in eq 2. Example ideal spectra and fits for C153 in neat glyceline are shown in the top panel of Figure 4. The resulting ν̃_0_(*t*) from the log-normal fits are then fit to a biexponential function with an offset for the equilibrium spectral position, ν̃_0_(*∞*):

$${\tilde{\nu }}_{0}(t)=a_{1}\exp(-t/\tau_{1})+a_{2}\exp(-t/\tau_{2})+{\tilde{\nu }}_{0}(\infty )$$ 6

and the integral solvation time calculated by

$$\tau_{\mathrm{solv}}=\frac{a_{1}\tau_{1}+a_{2}\tau_{2}}{a_{1}+a_{2}}$$ 7

Measured *s*(*t*) functions are shown in the middle panels of Figure 4 for both C153 and C343, integral solvation times are shown in the bottom panel of Figure 4, and biexponential fit parameters of ν_0_(*t*) are shown in Table 2. We note that choosing to fit *s*(*t*) with a biexponential does not (in this case) imply the presence of two independently relaxing populations, as would be assumed in traditional chemical kinetics. Solvation dynamics are the consequence of collective rotations and translations of all solvent molecules in the vicinity of the probe, which results in nonexponential dynamics.^40^ The biexponential function chosen here simply provides enough flexibility for the fit, and we will not interpret *a*_*i*_ or τ_*i*_, only the integral solvation time τ_solv_ plotted in the bottom panel of Figure 4.

**Figure 4:**
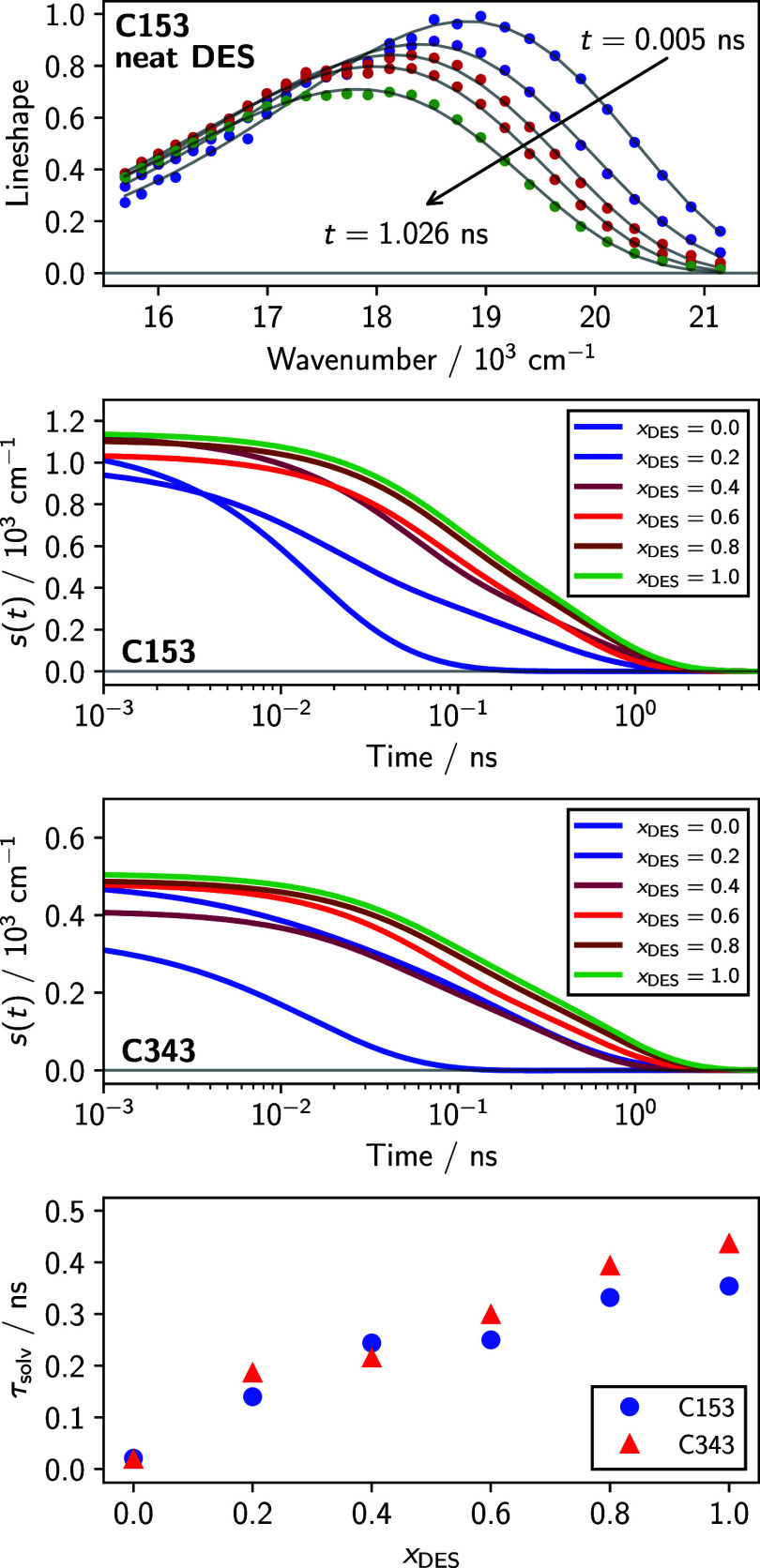
Top panel: time-resolved emission spectra of C153 in glyceline at 295 K. The points are from reconstruction data, while the lines are fits of these data to a log-normal function. Middle panels: time-dependent peak frequency shifts for all mole fractions. Bottom panel: observed integral solvation times for C153 (circles) and C343 (triangles) as a function of mixture composition. We estimate an uncertainty of ∼10% for these data, given the observed scatter.

**Table 2:** Fit Parameters for Fitting ν̃_0_(*t*) to Eq 6 for C153 and C343^a^

C153									
*x*_DES_	ν̃_0_(*∞*)	Δν̃_0_^obs^	*a*_1_	τ_1_	*a*_2_	τ_2_	τ_solv_	window	IRF fwhm
0.0	18.17	1.07	0.635	0.011	0.438	0.036	0.021	0.5	0.0085
0.2	17.68	0.95	0.522	0.018	0.482	0.288	0.140	1	0.0145
0.4	17.64	1.12	0.636	0.048	0.484	0.502	0.244	2	0.0264
0.6	17.63	1.04	0.470	0.062	0.571	0.405	0.250	5	0.0505
0.8	17.67	1.11	0.512	0.079	0.598	0.548	0.332	5	0.0544
1.0	17.72	1.14	0.496	0.080	0.646	0.564	0.354	5	0.0544

C343									
*x*_DES_	ν̃_0_(*∞*)	Δν̃_0_^obs^	*a*_1_	τ_1_	*a*_2_	τ_2_	τ_solv_	window	IRF fwhm
0.0	20.49	0.33	0.127	0.060	0.207	0.027	0.019	0.5	0.0085
0.2	20.22	0.46	0.166	0.020	0.296	0.280	0.186	0.5	0.0089
0.4	20.05	0.41	0.165	0.039	0.246	0.332	0.215	5	0.0505
0.6	20.03	0.48	0.232	0.062	0.250	0.518	0.299	5	0.0505
0.8	20.03	0.49	0.207	0.073	0.283	0.627	0.393	5	0.0505
1.0	20.01	0.51	0.201	0.073	0.305	0.675	0.436	5	0.0544

a The frequencies and amplitudes are given in units of 10^3^ cm^–1,^ and time constants are given in ns. The quantity Δν̃_0_^obs^ is the observed magnitude of the time-resolved Stokes shift and is determined by Δν̃_0_^obs^ = *a*_1_ + *a*_2_. Also included are the time windows over which the data were collected and the IRF fwhm, both reported in units of ns.

To further support the nonexponential nature of the solvation dynamics, we also fit ν̃_0_(*t*) to a single stretched exponential function, the results of which are shown in Section S1 of the Supporting Information. Stretched exponential functions model ν̃_0_(*t*) as a distribution of time-constants and use fewer fitting parameters than the biexponential. The use of the stretched exponential does not significantly degrade the quality of the fit, supporting our assertion that the spectral dynamics we observe are distributed and not the result of distinct populations of relaxing chromophores.

Both solvents display increases in τ_solv_ as a function of increasing glyceline concentration, as would be expected based on the increasing viscosity of the system.^30^ The *s*(*t*) functions that we measure are smoothly varying with time but are missing a large portion (over 50% in most cases) of the total shift due to the limited time resolution of the instrument. The missing portion of the dynamics corresponds to a very fast inertial component that dominates at short time which has been observed in dipolar solvents,^40^ ionic liquids,^45,46^ and deep eutectics^24^ previously. There are no obvious discontinuities in τ_solv_ as a function of *x*_DES_, and both probes exhibit roughly similar solvation times. This observation suggests that the solvation dynamics of these large probe molecules is somewhat independent of probe identity and that there are no large-scale changes in the environment they experience as the solvent composition is varied.

### 3.3 Solute Rotational Dynamics

Another common characterization method of the local solvent environment is the rotational dynamics of the solute. Here, we use fluorescence anisotropy measurements of C153 and C343 to probe these dynamics. This is accomplished by measuring TRE spectra with the emission polarizer rotated either parallel or perpendicular and at the magic angle with respect to the excitation pulse. These three measurements are related by the following expressions^47^:

$$\begin{array}{c} I_{∥}(t,\lambda )=\frac{1}{3}I(t,\lambda )[1+2r(t)]\\ I_{⊥}(t,\lambda )=\frac{1}{3}I(t,\lambda )[1-r(t)] \end{array}$$ 8

where *I*(*t*, λ), *I*_∥_(*t*, λ), and *I*_⊥_(*t*, λ) are the magic angle, parallel, and perpendicular emission decays at wavelength λ, respectively, and *r*(*t*) is the anisotropy. The anisotropy is directly related to the second-rank rotational time-correlation function of the chromophore, *C*_rot_^(2)^(*t*), according to

$$r(t)=r_{0}C_{\mathrm{rot}}^{\left(2\right)}(t)$$ 9

where

$$C_{\mathrm{rot}}^{\left(2\right)}(t)=\frac{3}{2}\langle \cos^{2}\;\theta (t)\rangle -\frac{1}{2}$$ 10

and

$$r_{0}=\frac{2}{5}\left(\frac{3}{2}\langle \cos^{2}\;\theta (0)\rangle -\frac{1}{2}\right)$$ 11

The θ­(*t*) is the angle between the absorption and emission transition dipole moments at time *t*, and *r*_0_ is the limiting anisotropy (the value of *r*(*t*) at *t* = 0). Both C153 and C343 have roughly parallel absorption and emission dipole moments; therefore, one expects *r*_0_ to approach the limiting value of 0.4. But, limited time resolution will serve to decrease *r*_0_ from its true value.

In this work, we do not calculate *r*(*t*) directly from emission intensities due to the high amount of noise in *r*(*t*) when using this method. Instead, we fit the three intensity decays simultaneously by assuming a sum of multi-exponentials for both *I*(*t*, λ) and *r*(*t*). This approach also allows us to directly calculate properly normalized χ^2^ values for each decay, which makes the evaluation of the quality of the fit straightforward. Sensitivity factors are also included to account for differing detector efficiencies, but were in all cases close to 1 due to the use of a polarization scrambler in the emission path. We validated our approach by comparison of *r*_0_ and τ_rot_ for C153 in a set of *n*-alcohols from the literature and with a time-correlated single photon counting apparatus as described in Section S2 of the Supporting Information. Three or four exponentials were used for modeling *I*(*t*), and only one was needed for *r*(*t*), whose time constant we take as the rotational correlation time, τ_rot_. Examples of such fits are given in Figure 5, and fitted values of *r*_0_ and τ_rot_ are shown in Figure 6 and reported in Table 3. Table 3 also includes predictions of τ_rot_ from hydrodynamic theory, which will be discussed in Section 4.

**Figure 5:**
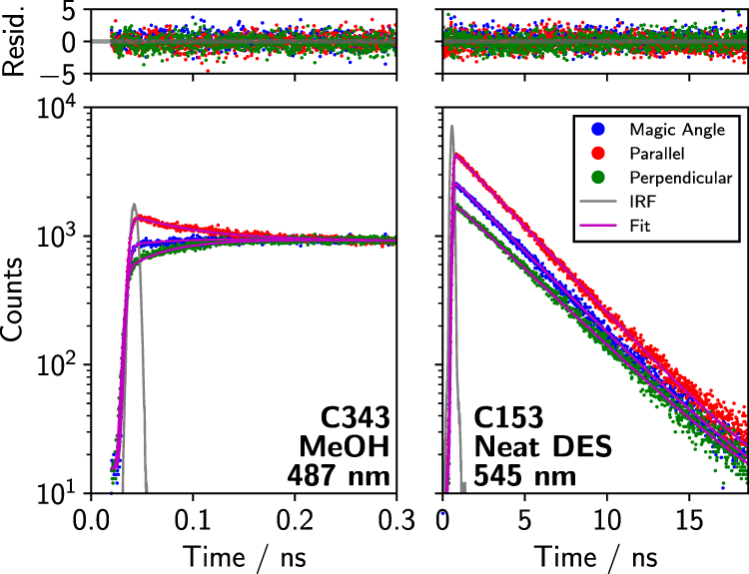
Polarized emission decays, fits, instrument response functions, and residuals for C343 in MeOH (left) and C153 in glyceline.

**Figure 6:**
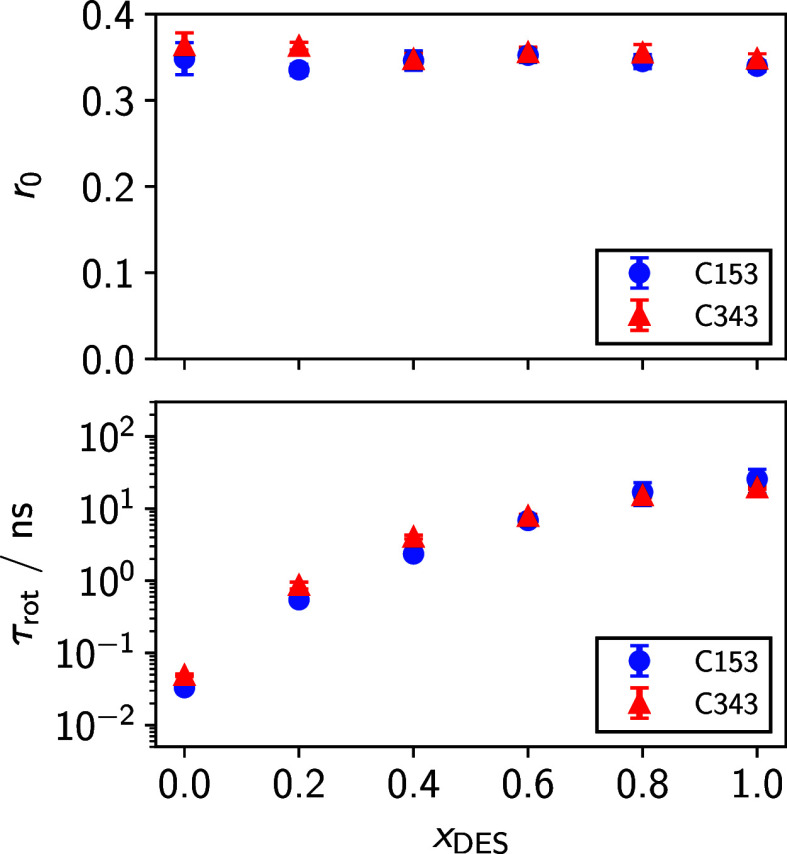
Initial anisotropies (top) and rotational correlation times (bottom) for C153 and C343 in the MeOH/glyceline mixtures. Error bars represent 2× the standard deviations of fits performed at 4 different wavelengths across the emission spectra.

**Table 3:** Fitting Parameters for the Fluorescence Anisotropy of C153 and C343 Figure 6 ^a^

				C153			C343		
*x*_DES_	η	τ_stk_	τ_slp_	*r*_0_	τ_rot_	*C*_obs_	*r*_0_	τ_rot_	*C*_obs_
0.0	0.58	0.060	0.014	0.348 ± 0.009	0.033 ± 0.002	0.55	0.364 ± 0.007	0.049 ± 0.001	0.82
0.2	3.20	0.330	0.079	0.335 ± 0.003	0.55 ± 0.03	1.65	0.363 ± 0.002	0.86 ± 0.05	2.62
0.4	13.8	1.422	0.341	0.346 ± 0.006	2.35 ± 0.05	1.65	0.348 ± 0.003	4.0 ± 0.1	2.84
0.6	49.0	5.047	1.211	0.352 ± 0.004	6.8 ± 0.3	1.36	0.356 ± 0.003	7.8 ± 0.2	1.55
0.8	162.5	16.76	4.021	0.345 ± 0.004	17 ± 3	1.01	0.355 ± 0.005	15.2 ± 0.5	0.91
1.0	516.1	53.20	12.77	0.340 ± 0.003	26 ± 5	0.48	0.348 ± 0.003	19.6 ± 0.5	0.37

a We fit four different wavelengths across the emission spectrum of each probe, and we report the average *r*_0_ and $\tau_{\mathrm{rot}}$ from these fits. Uncertainties are 2× the standard deviation of the four fits. The quantities τ_stk_ and τ_slp_ represent predictions of τ_rot_ from molecular hydrodynamics and *C*_obs_ is the observed rotational coupling constant as defined in eq 13. The viscosity is taken from ref 30. and is reported in mPa s. All times are reported in units of ns.

We find that both *r*_0_ and τ_rot_ are nearly identical for C153 and C343 as a function of the mixture composition. Rotation times monotonically increase with increasing glyceline concentration due to the increase in mixture viscosity. This observation suggests, as did the steady-state spectra, that C153 and C343 experience similar environments in the mixtures. In no case did we need more than a single exponential to describe *r*(*t*). This finding suggests that rotations of C153 and C343 are not sensitive to whatever temporal or environmental heterogeneities may be present in the mixtures. Although reports of heterogeneous solute dynamics in DESs are common,^10,22^ single-exponential rotational dynamics have been observed for fluorescence probes in these solvents,^48^ and in this solute/solvent pair specifically.^21^

## 4 Discussion

Both solvation dynamics and solute rotations can be related to diffusive motions of the solvent and solute therefore, we choose to discuss our results in the context of molecular hydrodynamics. For solvation, translations and rotations of the solvent around the solute modulate the local electric field drives the observed spectral relaxation. Solute rotations can also be modeled as a diffusive process in the Stokes–Einstein–Debye (SED) model, where the solute moves in small diffusive steps whose rate is controlled by the viscosity of the solvent. If the solute is modeled as an asymmetric ellipsoid, solute rotation times can be predicted using

$$\tau_{\mathrm{rot}}=\frac{fCV\eta }{k_{B}T}$$ 12

where *f* is a shape factor, *C* is the coupling constant (related to the degree of solute/solvent association), *V* is the volume of the solute, η is the solvent’s viscosity, and *T* is the temperature. While *f* is determined by the shape of the ellipsoid used to model the chromophore, *C* must be determined by solving the Navier–Stokes equations using one of two boundary conditions termed ‘stick’ and ‘slip’.^49^ The stick condition assumes that the velocity of the solvent at the surface of the rotor is the same as the rotor itself (i.e., the first layer of solvent ‘sticks’ to the rotor), whereas the slip condition assumes the solvent velocity is 0 at the rotor’s surface (i.e., the rotor ‘slips’ past the solvent). Under the stick condition, *C* = 1, whereas under slip boundary conditions, *C* must be determined from the ratio of the ellipsoid semiaxes.^50,51^ In our case, we model C153 and C343 using the same ellipsoid dimensions, and predictions for their rotational correlation times under stick and slip boundary conditions are given in Table 3. The parameters for the hydrodynamic predictions were taken from ref 52. The predictions from stick conditions will be termed τ_stk_ and slip as τ_slp_.

The SED equation is derived from molecular hydrodynamics, which assumes the solute is diffusing within a continuum environment described only by its viscosity. A key prediction from this model is that rotation times (or any correlation time that depends on diffusive motion, such as τ_solv_) are expected to be proportional to (η*T* ^–1^)^*p*^ with *p* = 1. If the solvation dynamics also follow molecular hydrodynamics, we would expect them to scale linearly with η*T* ^–1^, but with a prefactor different from that for rotations. Any deviations from *p* = 1 or nonpower law dependences of correlation time on η*T* ^–1^ suggest that the interaction between the solute and its local environment is changing, more than viscosity is needed to describe the solute/solvent coupling. We plot both τ_solv_ and τ_rot_ vs η*T* ^–1^ in the top panel of Figure 7, along with stick and slip predictions for τ_rot_. To quantify the deviation of the observed rotation times from the hydrodynamic predictions, we calculate a relative coupling constant, *C*_obs,_ by

$$C_{\mathrm{obs}}=\frac{\tau_{\mathrm{rot}}}{\tau_{\mathrm{stk}}}$$ 13

**Figure 7:**
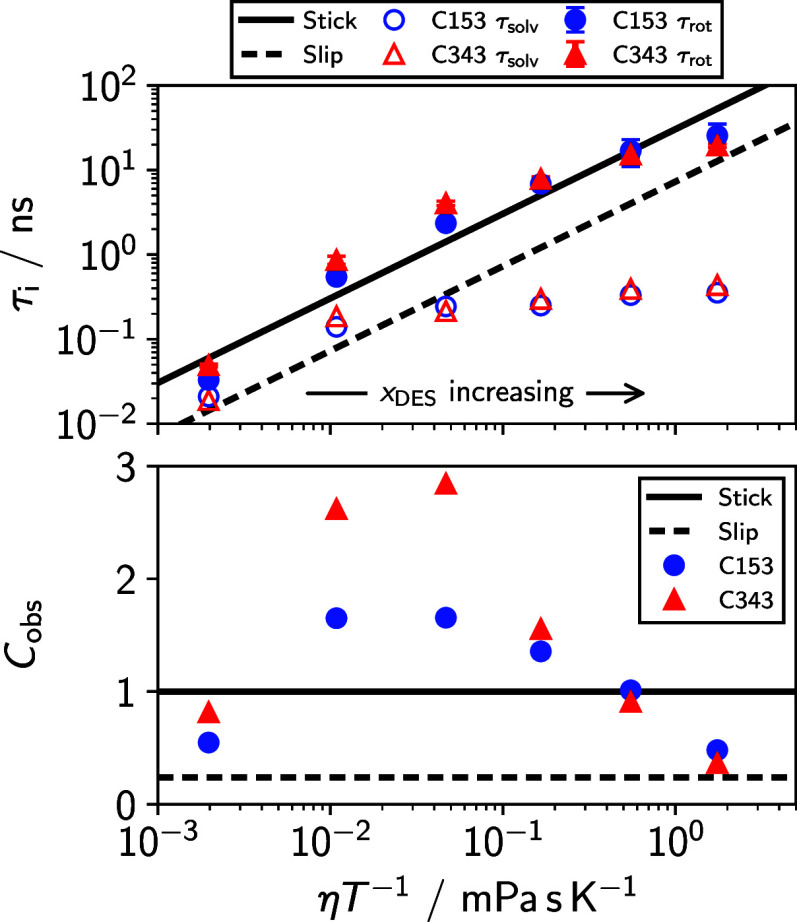
Top: integral rotation (filled symbols) and solvation (empty symbols) times for C153 and C343 plotted vs η*T* ^–1^. Bottom: rotational coupling constants for C153 and C343 calculated according to eq 13.

We see in the top panel of Figure 7 that neither τ_rot_ nor τ_solv_ strictly follows hydrodynamic predictions. The solvation times show a marked increase when glyceline is first introduced (going from *x*_DES_ = 0.0 to 0.2), then weakly increase as the mixtures become more viscous up to the introduction of more glyceline. Note that we do not expect τ_solv_ to follow either slip or stick hydrodynamic predictions, as those predictions are only for solute rotation, but we include them in Figure 7 to demonstrate their strongly nonhydrodynamic scaling. Fitting just the *x*_DES_ = 0.2–1.0 data to a power law yields a power of *p* = 0.18, significantly lower than the value of *p* = 1 predicted by molecular hydrodynamics. Such a small value of *p* is striking and smaller than usual, but powers substantially less than one are not unheard of in the context of solvation in DESs.^18,26,33,53–56^ We note that our small *p* is not likely an artifact, as our solvation times in neat methanol and glyceline are in strong agreement with literature values^21,40^ and previous studies of ethaline/methanol mixtures^33^ can also be shown to exhibit values of *p* < 0.30. Our particularly small *p* likely reflects the different mechanisms for changing viscosity due to changes in mixture composition rather than temperature. Another explanation could be that solvation dynamics, being driving by collective solvent rotations and translations, are coupled to viscosity differently than diffusive processes dependent on only one type of small-step motion.

The rotational correlation times of C153 and C343 do not follow a power law with respect to η*T* ^–1^. In neat MeOH τ_rot_ falls between the stick and slip predictions, becomes superstick for *x*_DES_ = 0.2, 0.4, and 0.6, then again falls to between stick and slip for *x*_DES_ = 0.8 and 1.0. This nonpower law dependence suggests that the coupling between the solute and solvent is changing dramatically as a function of mixture composition. The value of *C*_obs_ (bottom panel Figure 7) reflects this change as well, peaking for the median mixture compositions. Additionally, *C*_obs_ is somewhat larger for C343 and C153 in these median mixtures, whereas they are roughly the same at the extremes. Other models of rotational dynamics, such as Gierer–Wertz^57^ and Dote–Kivelson–Schwartz,^58^ could be used to predict τ_rot_, but would not be sufficient for describing these data. These other models change the prediction of *C* by incorporating solvent free volume or relative solute/solvent size, but still predict a (fractional) power law dependence of τ_rot_ on η*T* ^–1^ which we do not observe here.

We hypothesize that these trends in τ_rot_ and *C*_obs_ are the result of preferential solvation of probe molecules by the components of glyceline compared to MeOH. Such preferential solvation by glyceline would cause the probes to experience stronger local friction compared to the bulk, which would be dominated by the less viscous MeOH. Additionally, strong association between the probes and glyceline components could result in a larger effective hydrodynamic volume as the probe drags glyceline components along as it rotates. The larger couplings for C343 compared to C153 can also be explained by this model. By replacing the −CF_3_ group in C153 with a carboxyl group in C343 there are more potential hydrogen bonding sites to coordinate with choline and glycerol, increasing the magnitude of probe/glyceline association and increasing the effective hydrodynamic friction or volume. Preferential solvation may also explain the small *p* for solvation, as the probe solvent environment may not be changing very significantly with changes in mixture composition. Preferential solvation can be consistent with the lack of heterogeneity (i.e., single exponential anisotropies) in the rotational dynamics. Although the probe environment may be different than that of the bulk, if all chromophores are preferentially solvated in the same way and the environment persists throughout the relaxation, then one would expect to see homogeneous rotations even in a solvent with environmental heterogeneities. Another possible explanation for the apparent lack of heterogeneous dynamics could be that C153 and C343 are too large to be sensitive to whatever environmental heterogeneities are present in these solvents. The dynamics of small gas molecules, such as CO_2_, could be more affected than the larger fluorescence probes employed here, making them better probes of solvent heterogeneity.

## 5 Conclusions

In this work, steady-state and time-resolved fluorescence spectroscopy were used to study the solvation and rotational dynamics of C153 and C343 in mixtures of MeOH and glyceline. The steady-state experiments suggest that the polarity of the environment felt by the probes does not change significantly across mixture composition. We find that both τ_solv_ and τ_rot_ slow down with increasing glyceline concentration, but are coupled differently to changes in bulk viscosity. In particular, the solvation dynamics of both probes are very weakly coupled to viscosity as evidenced by a weak power-law dependence of τ_solv_ on η*T* ^–1^, exhibiting *p* ≪ 1. Fluorescence anisotropy experiments and their result τ_rot_ suggest the presence of preferential solvation and aggregation of glyceline components around the probe molecules, resulting in a non-power law dependence of τ_rot_ on η*T* ^–1^. Contrary to other reports, we did not observe indications of environmental heterogeneity, as all rotation times could be fit by single exponentials. Both the solvation and rotational dynamics experiments show the limitations of the streak camera time-resolution. Future studies will apply a protocol of merging time-ranges to maximize the time-resolution of the fast dynamics while still being able to resolve slower decay components. Molecular dynamics simulations of these systems are also planned in order to explore the molecular details of the solvation environment and possible preferential solvation.

## Supplementary Material



## Notes

Lineshape spectra, sample TRE fits, fits of the solvation correlation functions to stretched exponentials, and details on the validation of the anisotropy fitting procedure (PDF)
