# Supplementary material for: Do Dipolar Cosolvents Mitigate Microheterogeneity in Deep Eutectic Solvents? Solvation Dynamics and Solute Rotations in Glyceline/Methanol Solutions
Source: J Phys Chem B. 2025 Dec 28;130(1):396–406. doi: 10.1021/acs.jpcb.5c06534 (PMC12794137; doi:10.1021/acs.jpcb.5c06534)
Supplement: Supplementary file 1 [file jp5c06534_si_001.pdf]

Supporting Information for: Do Dipolar Cosolvents Mitigate Microheterogeneity in Deep Eutectic Solvents? Solvation Dynamics and Solute Rotations in Glyceline/Methanol Solutions

Christian Green,[†] Christopher A. Rumble,[*,‡] and Mark P. Heitz[*,†]

†*SUNY Brockport, Department of Chemistry and Biochemistry, Brockport, NY, 14420*

‡*The Pennsylvania State University – Altoona College, 3000 Ivyside Park, Altoona, PA 16601*

E-mail: crumble@psu.edu; mheitz@brockport.edu

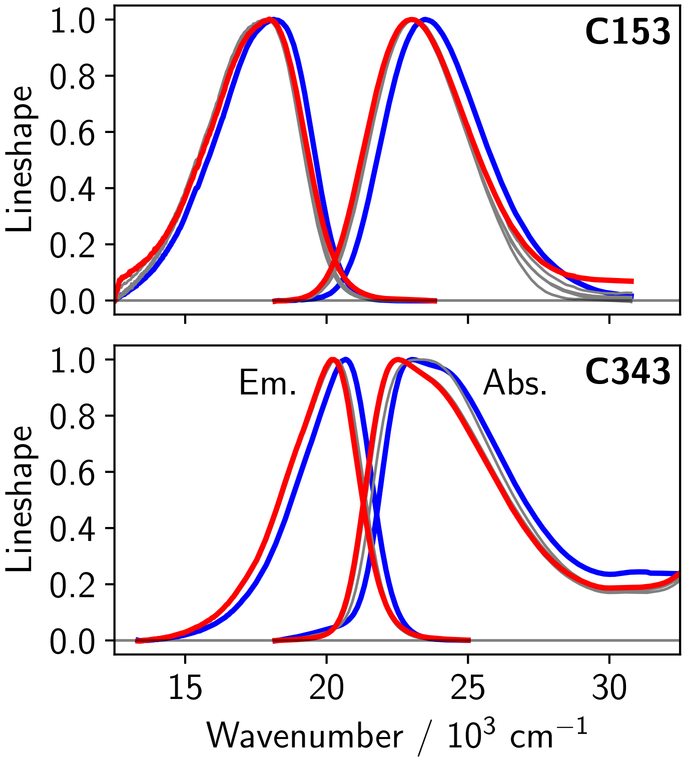

Figure S1: Steady-state absorption and emission lineshapes for C153 and C343.

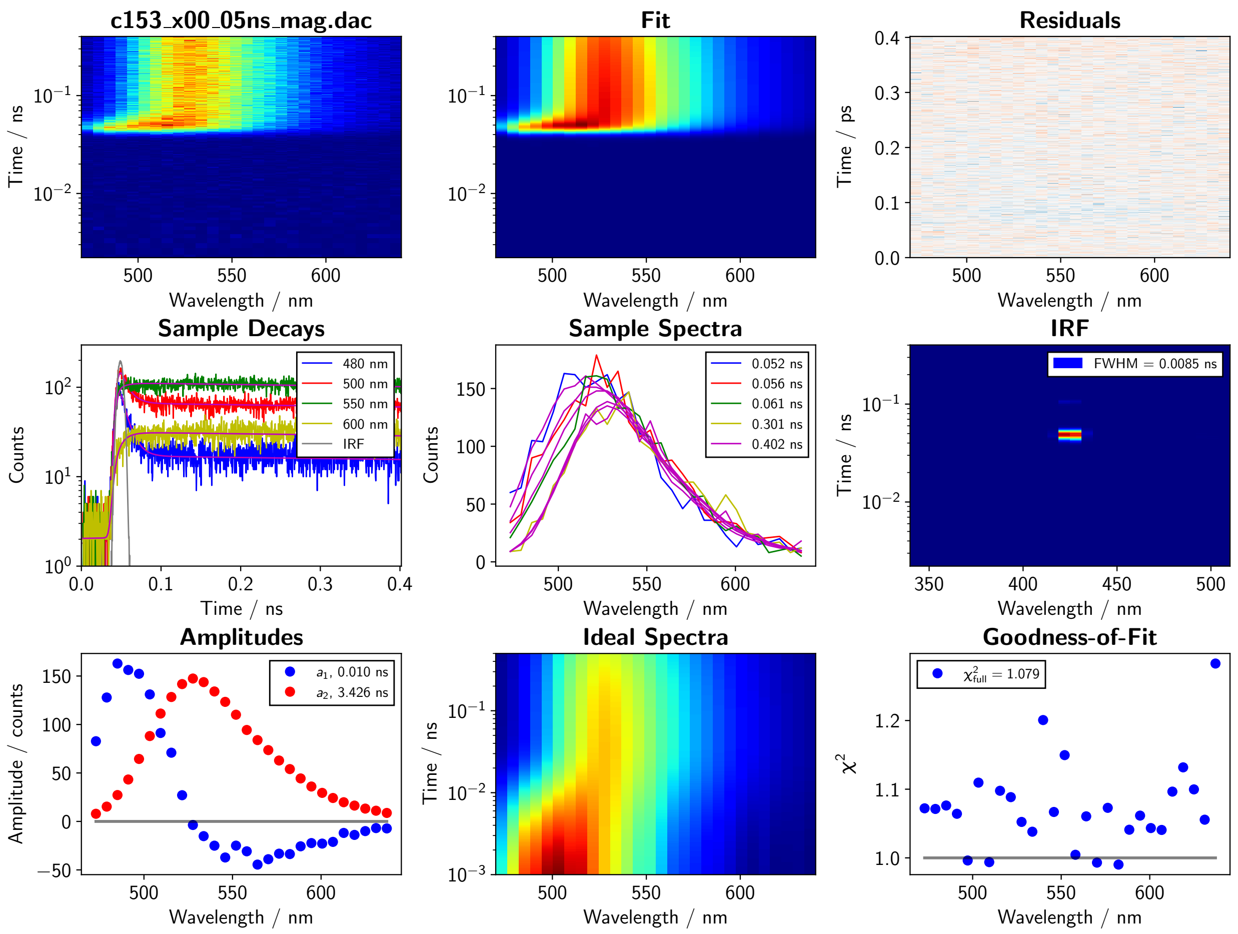

Figure S2: Example convolute-and-compare fit for C153 in neat MeOH collected over a 0.5 ns window.

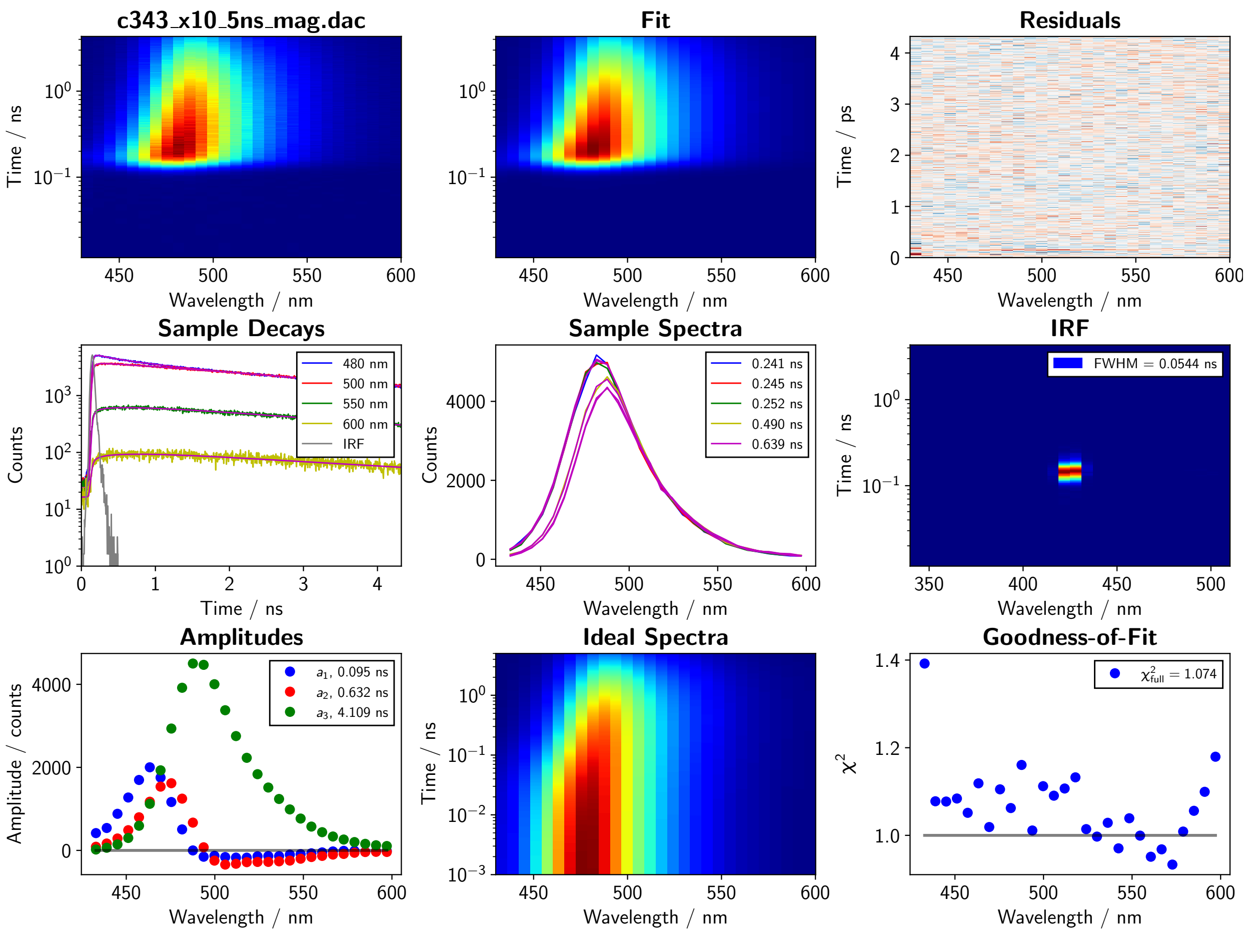

Figure S3: Example convolute-and-compare fit for C343 in neat glcyeline collected over a 20 ns window.

Section S1 Stretched Exponential Fits For Solvation Dynamics

We attempted to fit $\tilde{\nu}_0(t)$ using both bi-exponentials (Equation 6 of the main text) and a single stretched exponential of the form:

$$\tilde{\nu}_0(t) = h \exp(-[t/\tau]^{\beta}) + \tilde{\nu}_0(\infty) \tag{S1}$$

with integral times calculated according to:

$$\tau_{\text{solv}} = \frac{\tau}{\beta}\Gamma(\beta^{-1}) \tag{S2}$$

where h is a height parameter related to the magnitude of the shift, τ a time constant, $\tilde{\nu}_0(\infty)$ the equilibrium peak frequency, Γ is the gamma function, and β the 'stretching parameter' where $\beta = 1$ represents a single exponential and progressively smaller β indicate progressively broader distributions of time constants. This function intrinsically captures heterogeneity in the dynamics of $\tilde{\nu}_0(t)$. These results are summarized in Figure S4 and Table S1.

Figure S4: Fits of $s(t)$ to bi-exponentials (left) and stretched exponentials (right). Colored lines represent the data and gray lines the fit.

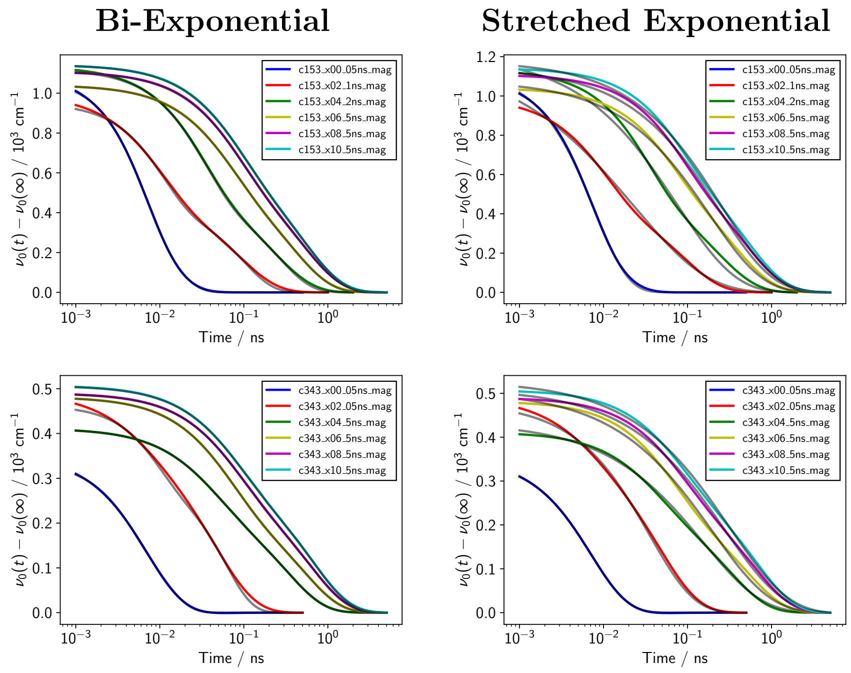

Table S1: Stretched exponential fitting parameters for $\tilde{\nu}_0(t)$. Times are reported in ns and h and $\tilde{\nu}_0(\infty)$ in 10^3 cm^{-1}.

	C153					C343				
x_{IL}	h	$\tilde{\nu}_0(\infty)$	τ	β	τ_{solv}	h	$\tilde{\nu}_0(\infty)$	τ	β	τ_{solv}
0.0	1.13	18.17	0.017	0.798	0.019	0.356	20.50	0.015	0.738	0.018
0.2	1.21	17.67	0.047	0.400	0.156	0.473	20.22	0.134	0.620	0.194
0.4	1.21	17.65	0.127	0.560	0.210	0.434	20.05	0.155	0.628	0.220
0.6	1.08	17.63	0.183	0.685	0.236	0.505	20.03	0.197	0.626	0.281
0.8	1.15	17.68	0.237	0.678	0.309	0.512	20.03	0.281	0.633	0.396
1.0	1.18	17.72	0.259	0.678	0.338	0.531	20.01	0.319	0.623	0.459

As seen in the gray lines (representing the fits) overlapping the colored lines (representing the solvation dynamics) in Figure S4, there is only a slight degradation of fit quality (by visual inspection) when using the stretched exponential model compared to the bi-exponential. Additionally, the fit parameters and τ_{solv} in Table S1 are nearly identical to those of the bi-exponential fits given in the main text. The ability to fit the dynamics with a function that contains intrinsically heterogeneous dynamics supports our assertion that the dynamics we observe are not the result of two distinct relaxing populations, as a bi-exponential in traditional chemical kinetics would suggest, but simply a distribution of relaxation rates. We choose to report the bi-exponential results in the main text as they are still higher quality fits than the stretched exponential.

Section S2 Anisotropy Fitting Validation

In order to validate the anisotropy experiment with the streak camera and data processing procedure, we measured the fluorescence anisotropy of C153 in neat MeOH, n-butanol (BuOH), n-pentanol (PeOH), and n-decanol (DeOH) with the streak camera system in the laboratory of MPH and the time-correlated single photon counting (TCPSC) experiment in the laboratory of CAR. The strong agreement in τ_{rot} between these two techniques gives us confidence in the non-hydrodynamic behavior of τ_{rot} seen in the MeOH/glyceline mixtures. Differences in r_0 are due to the limited time-resolution the the 20 ns window of the streak camera compared to TCSPC, whose time-resolution depends primarily on the response time of the photon counter and not measurement window.

Table S2: Anisotropy parameters for C153 in selected alcohols. All times are reported in ns.

Source	Solvent	Window	IRF	r_0	a_1	τ_1	a_2	τ_2	τ_{rot}
TCSPC	BuOH	25	0.024	0.280	0.121	0.070	0.160	0.387	0.250
Streak	BuOH	20	0.230	0.203	0.203	0.246	–	–	0.246
TCSPC	PeOH	25	0.031	0.287	0.141	0.095	0.145	0.546	0.324
Streak	PeOH	20	0.230	0.206	0.206	0.379	–	–	0.379
TCSPC	DeOH	25	0.031	0.291	0.157	0.235	0.134	1.857	0.960
Streak	DeOH	20	0.230	0.319	0.127	0.128	0.192	1.401	0.949
